# Sea-Sickness

**Published:** 1889-10-26

**Authors:** 


### Therapeutics.

### SEA-SICKNESS.

Dr. George Parsons, surgeon in the Ocean Steamship Company's service, recommends in the *Provincial Medical Journal* for October the use of antipyrin in the treatment of sea-sickness. Dr. Parsons states that after an extended trial he has found bromides, bismuth, chloral, cocaine, nitrite of amyl, etc., all more or less useless. But he has employed antipyrin for both the prophylaxis and treatment of sea-sickness with considerable success, as compared with all former experiences. His plan is to administer five grains of antipyrin, freshly dissolved in a wineglassful of water, at the time of embarking, and to repeat the dose in four hours. As a rule, three doses at most are required. Dr. Parsons further states that in these cases when nothing can be retained on the stomach, the antipyrin may be administered hypodermically. Now, after a considerable experience of sea-sickness, we have come to the conclusion that what benefits one person does no good to another, and that a remedy for sea-sickness has yet to be discovered. In the meantime, the best plan to escape sea-sickness is to open the bowels well before going on board. Holding in recollection that antipyrin not unfrequently excites unpleasant symptoms, and that there are persons who cannot take this drug, we are of opinion that great caution should be exercised in administering it to persons suffering from the depression caused by sea-sickness.

